# Arteriovenous Malformation *MAP2K1* Mutation Causes Local Cartilage Overgrowth by a Cell-Non Autonomous Mechanism

**DOI:** 10.1038/s41598-020-61444-x

**Published:** 2020-03-10

**Authors:** Dennis J. Konczyk, Jeremy A. Goss, Patrick J. Smits, Christopher L. Sudduth, Alyaa Al-Ibraheemi, Arin K. Greene

**Affiliations:** 1 Department of Plastic & Oral Surgery, Boston Children’s Hospital, Harvard Medical School, Boston, USA; 2 Department of Pathology, Boston Children’s Hospital, Harvard Medical School, Boston, USA

**Keywords:** Disease genetics, Cartilage development

## Abstract

Extracranial arteriovenous malformation (AVM) is most commonly caused by *MAP2K1* mutations in the endothelial cell. The purpose of this study was to determine if local tissue overgrowth associated with AVM is caused by direct or indirect effects of the *MAP2K1* mutation (i.e., cell-autonomous or cell-non autonomous). Because cartilage does not have blood vessels, we studied ear AVMs to determine if overgrown cartilage contained AVM-causing mutations. Cartilage was separated from its surrounding tissue and isolated by laser capture microdissection. Droplet digital PCR (ddPCR) was used to identify *MAP2K1* mutations. *MAP2K1* (p.K57N) variants were present in the tissue adjacent to the cartilage [mutant allele frequency (MAF) 6–8%], and were enriched in endothelial cells (MAF 51%) compared to non-endothelial cells (MAF 0%). *MAP2K1* mutations were not identified in the overgrown cartilage, and thus local cartilage overgrowth likely results from the effects of adjacent mutant blood vessels (i.e., cell-non autonomous).

## Introduction

Arteriovenous malformation (AVM) is a fast-flow, congenital vascular anomaly that is locally destructive and characterized by abnormal connections between arteries and veins. Most lesions are sporadic and enlarge to cause significant morbidity. AVMs often involve multiple tissue planes and cause overgrowth of skin, bone, muscle, and cartilage^1,2^. Treatment includes embolization or resection; drugs for AVM do not exist. We previously reported that extracranial AVMs contain somatic mutations in *MAP2K1* that are isolated to endothelial cells (ECs)^3^. The mechanism by which AVMs cause overgrowth of involved tissues is unknown. The purpose of this study was to determine if tissue overgrowth associated with AVM is caused by direct or indirect effects of a *MAP2K1* mutation (i.e., cell-autonomous or cell-non autonomous). Understanding the mechanism by which AVMs enlarge may lead to the development of pharmacotherapy for patients.

## Results

**Figure 1:**
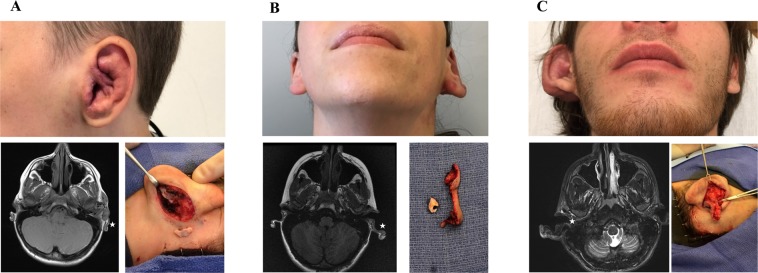
Study cohort of patients with auricular AVMs. All subjects have diffuse AVMs involving all components of the entire ear (i.e., skin, subcutaneous adipose, cartilage). MRI shows enlarged cartilage (low signal, stars). Intraoperative images (panels A and C) illustrate overgrown conchal cartilage that was removed as part of an otoplasty procedure to improve the appearance of the ear. Intraoperative photo for panel B shows separation of excised cartilage from surrounding skin and subcutaneous tissue. A = Patient 1. B = Patient 2. C = Patient 3.

**Figure 2:**
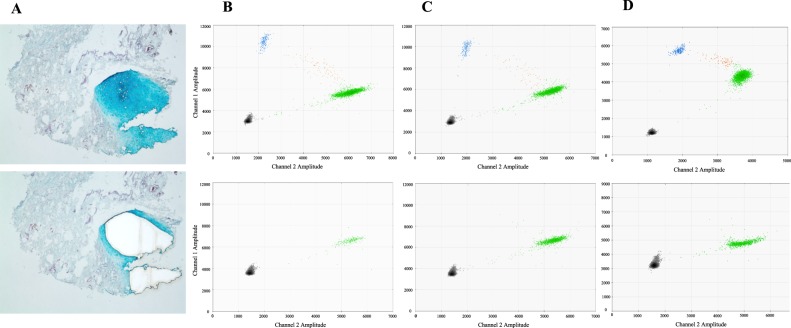
Somatic *MAP2K1* mutations are isolated to the skin and subcutis of ear AVM tissue. (**A**) Laser capture microdissection of cartilage from surrounding tissue to minimize inclusion of adjacent microscopic vessels containing mutant endothelial cells (Alcian Blue stain; Patient 2). Top panel = pre-microdissection, bottom panel = post-microdissection. **B**,**C**,**D** = Patient 1, 2, 3 ddPCR graphs of their AVM ear tissue. Top row of graphs = skin and subcutaneous adipose. Bottom row of graphs = cartilage. Left upper blue droplets contain mutant alleles. Right middle orange droplets have mutant and wild-type alleles. Right lower green droplets contain wild-type alleles. Left lower black droplets are empty. Note absence of mutant droplets in the cartilage graphs.

**Figure 3:**
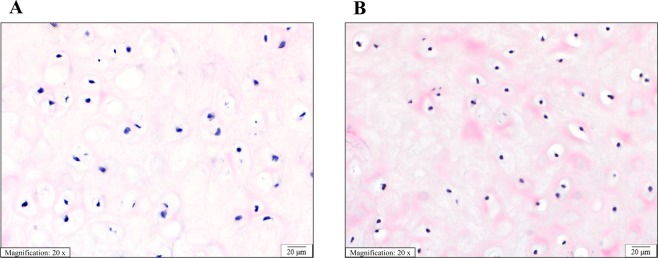
Histological appearance of overgrown AVM cartilage and normal cartilage is similar. Sections of (**A**) conchal ear cartilage from a patient with an AVM (Patient 3). (**B**) Control conchal ear cartilage from a patient with a normal ear. Sections show equivalent distribution and cellularity of chondrocytes in a chondromyxoid matrix. The chondrocytes have normal appearance with monomorphic pyknotic nuclei. (Hematoxylin and eosin stain, 20x magnification, scale bar 20 µm).

Three patients had an auricular AVM causing enlargement of all structures of the ear: Patient 1 (11 year-old male), Patient 2 (18 year-old female), Patient 3 (21 year-old male) (Fig. 1). M*AP2K1* (p.K57N) mutations were found in the tissue adjacent to the cartilage (i.e., skin and subcutaneous adipose) in all patients; the mutant allele frequency (MAF) was 6–8% (Table 1, Fig. 2). The mutation was enriched in ECs (MAF 51%) compared to non-ECs (0%). A *MAP2K1* mutation was not identified in 1 cartilage specimen, and the other 2 cartilage specimens had a MAF of 0.2% and 0.1% that we considered background noise^4^. Overgrown AVM ear cartilage in all 3 patients appeared similar to normal ear cartilage histologically. No difference was found between proteoglycan, elastin, type 6 collagen, or type 2 collagen; the cartilage also contained the same chondrocyte and extracellular matrix density and relationship (Fig. 3).

**Table 1:** Study Cohort.

Patient	Mutation	Skin/Subcutaneous Tissue (MAF)	Cartilage (MAF)	ECs/Non-ECs (MAF)
1	MAP2K1 (p.K57N)	7.8% (235/3031)	0.2%^*^ (12/5400)	—
2	MAP2K1 (p.K57N)	6.2% (545/8770)	0.0% (0/446)	—
3	MAP2K1 (p.K57N)	8.2% (340/4137)	0.1%^*^ (4/3275)	51.0%/0.0%

MAF (mutant allele frequency) = mutant droplets/total droplets (mutant + wild-type).(−) not performed. EC = endothelial cell. ^*^Considered background noise^4^.

## Discussion

Somatic mutations for many types of vascular anomalies recently have been described^5^. However, the mechanism by which these mutations cause vascular anomalies and contribute to their enlargement remains unknown. Extracranial AVM progresses over time and causes overgrowth of tissues, including skin, subcutis, muscle, cartilage, and bone^1,2^. We previously have shown that extracranial AVMs contain somatic *MAP2K1* mutations that are only contained in endothelial cells^3^. Because AVMs involving the ear are associated with significant cartilage overgrowth^1^ and cartilage does not contain vasculature^6^, we studied this clinical scenario to gain insight into the pathophysiology of AVMs.

Our data shows that only the vascularized tissue adjacent to cartilage of auricular AVM contains somatic *MAP2K1* mutations; the underlying overgrown cartilage does not. Consequently, the enlargement of cartilage does not result directly from a mutation in the cartilage (cell-autonomous). Instead, cartilage hypertrophy occurs secondarily to its surrounding soft tissue containing a vasculature with mutant *MAP2K1* endothelial cells (cell-non autonomous).

The histological appearance of the AVM cartilage was no different than normal cartilage. This finding may occur because AVM and cartilage enlargement occurs slowly over many years. Since the overgrowth is gradual, the chondrocyte and extracellular matrix density and protein expression may have time to equilibrate and appear similar to normal cartilage. One potential hypothesis to explain AVM-associated cartilage overgrowth could be paracrine effects from neighboring mutant cells (e.g., secretion of growth factors). Another possibility might be that increased pressure of adjacent tissues on the cartilage causes enlargement. The local vascular environment also may influence the overgrowth of cartilage. Increased blood flow and/or local ischemia causing reactive neovascularization may promote enlargement. However, capillary malformation, venous malformation, and lymphatic malformations, which do not exhibit increased arterial flow or shunting/ischemia, also cause underlying cartilage and bony hypertrophy^7–9^.

In contrast to AVM, which is present at birth and slowly enlarges over time, overgrowth conditions that are fully penetrant at birth and do not exhibit significant postnatal enlargement contain somatic mutations in all tissues. For example, facial infiltrating lipomatosis and CLOVES syndrome (congenital lipomatous overgrowth with vascular, epidermal, and skeletal anomalies) are caused by somatic *PIK3CA* mutations and exhibit overgrowth of every tissue (i.e., skin, subcutis, muscle, bone, cartilage)^10,11^. These conditions contain *PIK3CA* mutations in multiple cell types and in all overgrown tissues^10,11^. Consequently, these disorders likely result from a mutation in a multipotent progenitor cell (cell-autonomous) that contributes to mesodermal derivatives (e.g. stroma, adipose, muscle, bone) as well as to ectodermal structures (e.g., nerves).

We predict that other major vascular malformations (capillary, lymphatic, venous) cause overgrowth of tissues by a similar cell-non autonomous mechanism as AVM. Causative *GNAQ* mutations in capillary malformations, like AVM, are enriched in endothelial cells^12^ and capillary malformation also results in overgrowth of soft-tissue, cartilage, and bone over time^7,12^. A dynamic interaction between mutant endothelial cells and other cell types is likely a more favorable paradigm for the development of pharmacotherapy compared to overgrowth conditions such as facial infiltrating lipomatosis which are static and contain mutations in all cell types. Drugs might be able to prevent the progression of AVM and other vascular malformations by blocking overgrowth of tissues (e.g., cartilage) that do not contain a somatic mutation.

## Methods

This study was approved by the Committee on Clinical Investigation at Boston Children’s Hospital, and the procedures followed were in accordance with the ethical standards of the Helsinki Declaration of 1975, as revised in 2008. Written informed consent was obtained from participants prior to inclusion in the study. Because auricular AVMs cause overgrowth of the cartilage^1^, and ear cartilage does not have blood vessels containing endothelial cells that can possess a somatic *MAP2K1* mutation^6^, we chose this unique anatomical site to study. We obtained cartilage and its overlying skin and subcutis during a clinically-indicated otoplasty procedure to improve the appearance of 3 patients with an AVM causing a prominent ear deformity. The cartilage was separated from its overlying tissue (i.e., skin, subcutis) and in one patient ECs were separated from non-ECs. Because *MAP2K1* mutations are known to be enriched in ECs, cartilage was subjected to laser capture microdissection to minimize inclusion of adjacent microscopic tissue that might contain blood vessels with a *MAP2K1* mutation. Genomic DNA was extracted from cartilage, skin/subcutaneous tissue, and isolated cells and tested for mutant *MAP2K1* alleles using droplet digital PCR (ddPCR).

Cell isolation was performed as previously described^3,11^. Ear tissue was washed in PBS to remove blood cell contaminants, digested with collagenase A (2.5 mg/mL) (Roche) for 1 hour at 37 °C, then filtered through a 100 µm strainer to produce a single cell suspension. Cells were placed on fibronectin-coated (1 µg/cm^2^) tissue culture plates (Olympus Plastics) in endothelial growth medium-2 (EGM-2, Lonza) supplemented with 10% fetal bovine serum (FBS, Gibco, Life Technologies). After 5–7 days of expansion, cells were fractionated into 2 populations (endothelial and non-endothelial) using anti-human CD31 (endothelial cell marker) magnetic beads (DynaBead, Life Technologies). DNA was extracted from each cell population using the DNeasy Blood & Tissue kit following the manufacturer’s instructions (Qiagen).

Cartilage from resected AVM tissue was frozen in embedding medium (Sakura), cut into 7 µm sections, and placed on membrane glass slides (ThermoFisher). To distinguish cartilage from non-cartilage tissue microscopically, slides were fixed in 10% formalin and stained with Alcian Blue for the cartilage specific proteoglycan aggrecan and Nuclear Fast Red (Sigma). Using a Zeiss PALM Combi Laser Capture Microscope cartilage was isolated from adjacent non-cartilage tissue. DNA extraction was performed using an Agencourt FormaPure DNA kit (Beckman Coulter) without deparaffinization. PCR primers, fluorescent probes, and methods for detecting wild-type and mutant *MAP2K1* alleles have been previously described^3^. For each ddPCR reaction we used 45 nanograms of template DNA, corresponding to ~6000 cells. ddPCR was performed using a QX200 Droplet Generator, QX200 Droplet Reader, and QuantaSoft Software (Bio-Rad).

Additional AVM cartilage sections were digested with chondroitinase ABC (Sigma Aldrich) for one hour at 37 °C in a humid chamber, blocked for one hour at room temperature, incubated with primary antibody (1:200 dilution in blocking buffer) overnight at 4 °C, and incubated with biotinylated secondary antibodies as well as fluorophore conjugated streptavidin at room temperature for 1 hour and 45 minutes respectively. Primary antibodies used were specific for Aggrecan (Sigma-Aldrich AB1031), Collagen Type II (Sigma-Aldrich MAB8887), Elastin (Abcam ab21610), and Collagen Type VI (Abcam ab6588). Other sections were stained with hematoxylin and eosin. Histology of AVM ear conchal cartilage was compared to normal ear conchal cartilage from a control patient who had ear cartilage removed as part of a cosmetic otoplasty procedure. Images were obtained at 20X magnification using Nikon Eclipse 80i microscope with SPOT RT3 camera and SPOT 5.2 software.

## Data Availability

All data generated or analyzed during this study are included in this published article.
